# Investigation of sugar binding kinetics of the *E. coli* sugar/H^+^ symporter XylE using solid-supported membrane-based electrophysiology

**DOI:** 10.1016/j.jbc.2021.101505

**Published:** 2021-12-18

**Authors:** Andre Bazzone, Laura Tesmer, Derya Kurt, H. Ronald Kaback, Klaus Fendler, M. Gregor Madej

**Affiliations:** 1 Department of Biophysical Chemistry, Max-Planck-Institute of Biophysics, Frankfurt/M, Germany; 2 Department of Physiology and Department of Microbiology, Immunology, Molecular Genetics, University of California, Molecular Biology Institute, Los Angeles, California, USA; 3 Institute of Biophysics and Biophysical Chemistry, Department of Structural Biology, University of Regensburg, Regensburg, Germany; 4 Institute of Biophysics, Department of Structural Biology, Saarland University, Center of Human and Molecular Biology, Homburg, Germany

**Keywords:** membrane transport, SSM, energy coupling, bioenergetics, sugar transporter, induced fit, MFS, XylE, BC, betweenness centrality, DDM, *n*-dodecyl-beta-d-maltoside, EHS, high-energy occluded intermediate (substrate-bound and protonated), FucP, fucose permease, LacY, lactose permease, LPR, lipid-to-protein ratio, MFS, major facilitator superfamily, PSS, pre steady-state, SSM, solid-supported membrane, TDG, β-d-galactopyranosyl-1-thio-β-d-galactopyranoside, XylE, *Escherichia coli* xylose permease

## Abstract

Bacterial transporters are difficult to study using conventional electrophysiology because of their low transport rates and the small size of bacterial cells. Here, we applied solid-supported membrane–based electrophysiology to derive kinetic parameters of sugar translocation by the *Escherichia coli* xylose permease (XylE), including functionally relevant mutants. Many aspects of the fucose permease (FucP) and lactose permease (LacY) have also been investigated, which allow for more comprehensive conclusions regarding the mechanism of sugar translocation by transporters of the major facilitator superfamily. In all three of these symporters, we observed sugar binding and transport in real time to determine *K*_*M*_, *V*_max_, *K*_*D*_, and *k*_obs_ values for different sugar substrates. K_*D*_ and *k*_obs_ values were attainable because of a conserved sugar-induced electrogenic conformational transition within these transporters. We also analyzed interactions between the residues in the available X-ray sugar/H^+^ symporter structures obtained with different bound sugars. We found that different sugars induce different conformational states, possibly correlating with different charge displacements in the electrophysiological assay upon sugar binding. Finally, we found that mutations in XylE altered the kinetics of glucose binding and transport, as Q175 and L297 are necessary for uncoupling H^+^ and d-glucose translocation. Based on the rates for the electrogenic conformational transition upon sugar binding (>300 s^−1^) and for sugar translocation (2 s^−1^ − 30 s^−1^ for different substrates), we propose a multiple-step mechanism and postulate an energy profile for sugar translocation. We also suggest a mechanism by which d-glucose can act as an inhibitor for XylE.

The exceptionally diverse major facilitator superfamily (MFS), one of the two largest families of membrane transporters found on earth, includes membrane transport proteins from *Archaea* to *Homo sapiens* (1, 2)*.* In many instances, the MFS transporters use the driving force stored as a bulk phase, transmembrane electrochemical ion gradient (Δ*μ̃*_H_+) for the accumulation of substrates against their concentration gradients (secondary active transport). The paradigm for MFS, lactose permease (LacY), catalyzes the coupled translocation of a galactoside and an H^+^ across the *Escherichia coli* membrane (galactoside/H^+^ symport) (3). The structure models of LacY and other MFS sugar/H^+^ symporters show two pseudosymmetrical 6-helix bundles forming a deep cavity that contains the substrate-binding sites at its apex (reviewed in Ref. (4)). A large number of biophysical and biochemical data confirms that the single substrate-binding site is reciprocally accessible from the periplasmic side (outward-facing state) or cytoplasmic side (inward-facing state) of the membrane (see for reviews, Refs. (5, 6)). Thus, providing experimental evidence for the hypothesis that the MFS members function according to an alternating-access mechanism (7). Remarkably, the imposition of Δ*μ̃*_H_+ does not affect the rates of equilibrium exchange or counterflow with LacY (8, 9, 10), suggesting that the conformational change resulting in alternating access of the sugar-binding and H^+^-binding sites to either side of the membrane is a consequence of sugar binding and dissociation, and not Δ*μ̃*_H_+ (3). In this regard, the X-ray crystallographic studies of single-Cys122 LacY with covalently bound MTS-Gal (11) and the conformationally trapped mutant cocrystallized with β-d-galactopyranosyl-1-thio-β-d-galactopyranoside (TDG) (12) or an α-substituted galactoside (13) as well as the structures of the human MFS uniporters, GLUT1 with β-nonylglucoside trapped in the binding site (14) and GLUT3 with bound d-glucose (15), indicate that only a fully liganded substrate affects the transition into the occluded state of the transporter. Based on structural and other observations, it has been postulated that sugar binding to LacY involves an induced fit causing the N-terminal and C-terminal bundles converge as given side chains from both the N-terminal and C-terminal helix bundles ligate the sugar (3, 12, 16, 17, 18, 19).

However, many transporters from the MFS and other families bind their substrates only weakly because this is a physical requirement for rapid transport. This creates a challenge for the respective transporter to discriminate between the correct substrate and chemically similar molecules. To understand the kinetic and conformational perturbations associated with sugar binding and induced fit in the bacterial homolog of the GLUT family, XylE, we have expressed, purified, and reconstituted three different bacterial sugar/H^+^ symporters (xylose/H^+^, XylE; lactose/H^+^, LacY; and fucose/H^+^, FucP) from *E. coli* into proteoliposomes and compared the electrophysiological properties of symport with different sugars using solid-supported membrane (SSM)-based electrophysiology (20, 21). The time resolution and sensitivity of this method allows the identification of different transport properties with different sugars that are attributed to the sugar binding and induced fit. Furthermore, the substrate selectivity of XylE and LacY is analyzed in an integrative approach concerning all residues of the transporter. Together with the kinetic findings, this analysis indicates that the substrates are identified beyond the level of the binding site of the transporter.

## Results

### Charge translocation upon sugar transport

**Figure 1:**
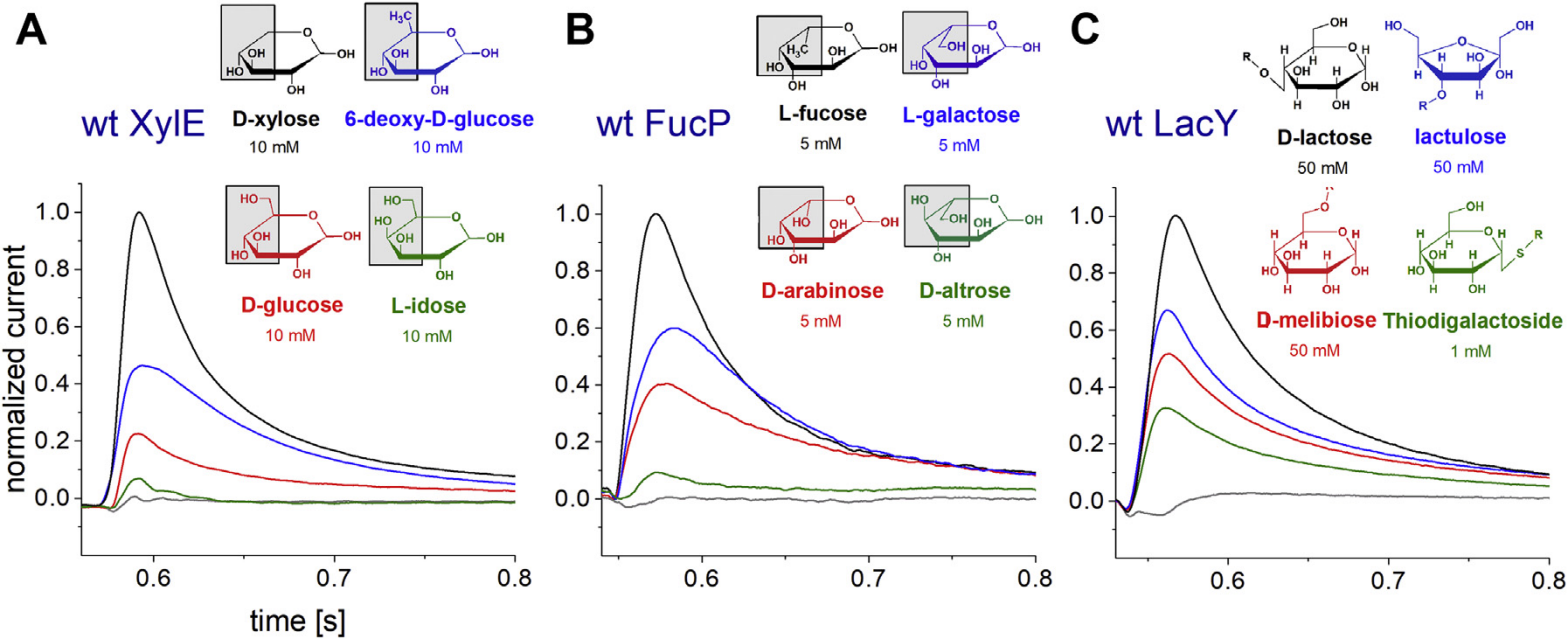
**Substrate specificity of transient currents.** For wt XylE (*A*, pH 7.0), wt FucP (*B*, pH 8.0), and wt LacY (*C*, pH 7.6). Current traces were induced by different substrate concentration jumps as indicated. The type of substrate is indicated by color; the *gray* trace is always the negative control; it results from a sucrose concentration jump of 10 mM (XylE), 5 mM (FucP), and 50 mM (LacY), respectively. All traces visualized within the same graph are recorded from the same sensor and normalized to the peak current of the substrate with the highest peak current. For comparison, the Haworth structures of the sugars are shown. The differences in the structures are highlighted in *gray squares*. R in the structures within *C* represents the galactoside residue of the illustrated disaccharides. The different peak currents for different substrates are either explained by different *K*_*M*_ or different *I*_max_ values (compare Fig. 2 and Table 1).

In SSM-based electrophysiology, the sugar/H^+^ symport activity of a transporter generates a transient current by capacitive coupling (22, 23). The peak of the transient current approximates the steady-state transport rate and will be therefore referred to as transport current. Different sugars were tested for the induction of currents with proteoliposomes containing one of the reconstituted sugar transporters XylE, FucP, or LacY (Fig. 1). Among the tested sugars, 6-deoxy-d-glucose as well as l-idose with XylE and d-altrose with FucP generated signals identifying them as substrates of the respective transporter (Table 1). Interestingly, the comparison of the transported sugars indicates that similar to LacY (12, 24), the presence and orientation of the OH group at position C4 is particularly critical for transport (Fig. 1).

**Table 1:** *I*_max_ and *K*_*M*_ values for all tested substrates and wt transporters

Transporter	pH	Substrate	*K*_*M*_ (mM)	*I*_max_
			Michaelis–Menten equation	
wt XylE	7.6	**d****-xylose**	1.7 ± 0.2	1.8 ± 0.5 nA
	7.6	6-Deoxy-d-glucose	0.2 ± 0.05	35 ± 5%
	7.6	d-glucose^a^	0.4 ± 0.1	26 ± 5%
	7.0	l-idose	—	7%^b^
wt FucP	7.6	**l****-fucose**	0.9 ± 0.1	0.5 ± 0.2 nA
	7.6	l-galactose	7.4 ± 1.3	123 ± 8%
	7.6	d-arabinose	12.2 ± 2.8	132 ± 13%
	8.0	d-altrose	—	9%^b^
wt LacY	7.6	**d****-lactose**	5.3 ± 0.5	0.7 ± 0.2 nA
	7.6	Lactulose	2.3 ± 0.6	71 ± 8%
	7.6	Melibiose	—	52%^b^
	7.6	TDG	0.12 ± 0.02	21 ± 1%

**Figure 2:**
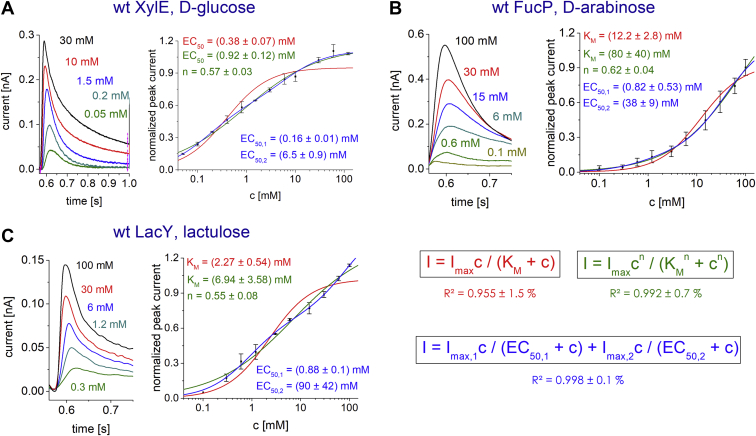
**Substrate dependence and determination of *K***_***M***_**values for different substrates.** With wt XylE (*A*), wt FucP (*B*), and wt LacY (*C*). *Left,* substrate-dependent transient currents. All traces visualized within the same graph are recorded from the same sensor. *Right,* substrate-dependent peak currents. All data points represent normalized and averaged values from n = 3 different sensors. Error bars reflect standard deviations. Before averaging datasets from different sensors, each dataset has been normalized to its *I*_max_ value. Michaelis–Menten (*red curve and equation*), Hill (*green curve and equation*), and double Michaelis–Menten (*blue curve and equation*) fits are shown. The average *R*^2^ for the three fitting curves are given below the respective equation. *K*_*M*_ values are obtained from concentration-dependent peak currents, when the current reflects transport; the *K*_*M*_ represents the half saturation constants for steady-state transport. For d-glucose instead of *K*_*M*_, the empirical half saturation constant EC_50_ is used because d-glucose does not induce full transport cycles in wt XylE (6, 31, 32) as discussed in the main text and shown in Figure 3*A*. The double Michaelis–Menten model assumes that pre steady-state (PSS) currents affect the peak current significantly, and therefore, two different EC_50_ values are measured, one for transport and one for the PSS reaction. The *K*_*M*_ values obtained from fits with the Michaelis–Menten equation and the *I*_max_ values of all tested substrates are summarized in Table 1. All measurements were performed at pH 7.6 using the low time-resolution set up.

**Figure 3:**
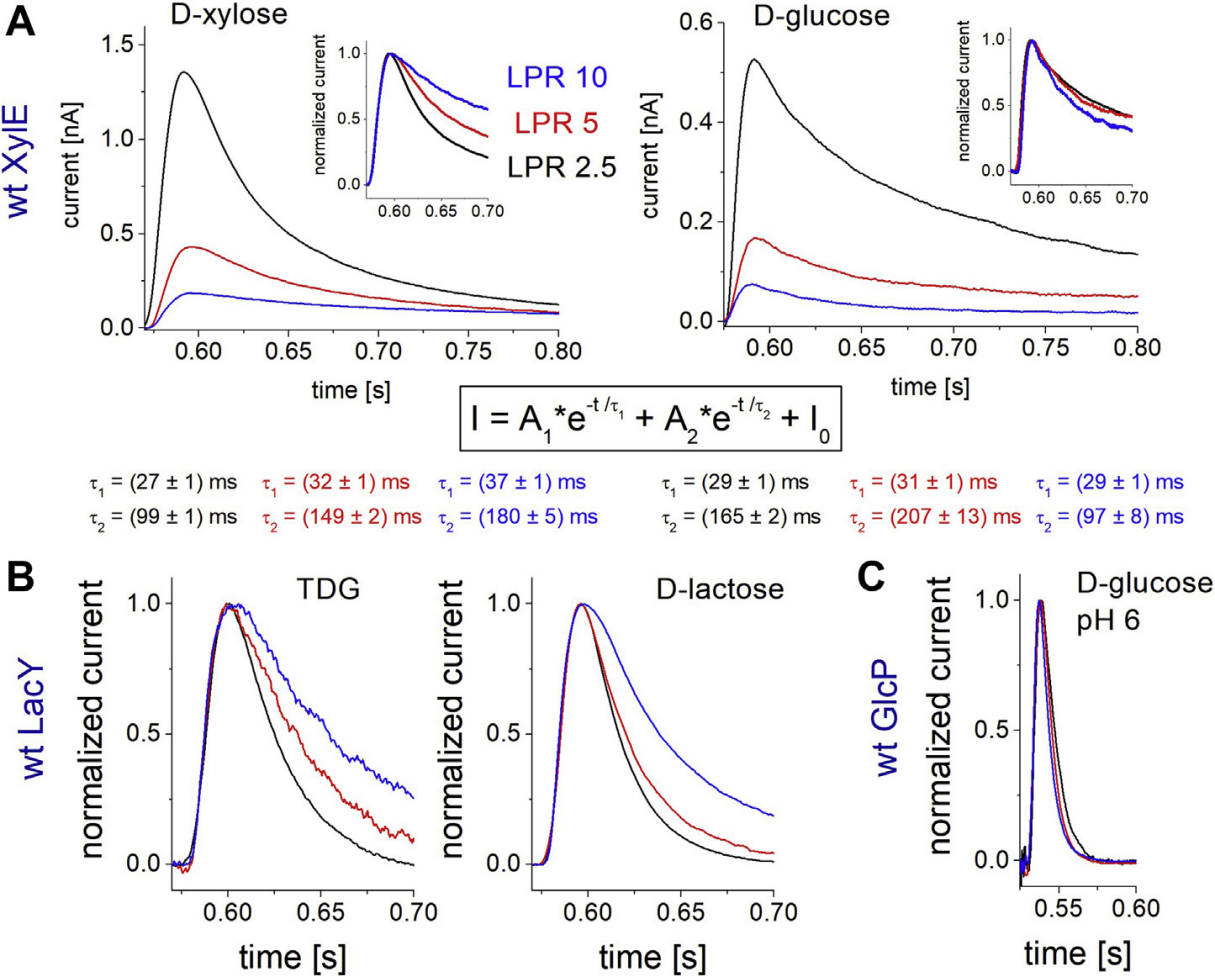
**Transient currents for wt transporters reconstituted at different lipid-to-protein ratios (LPRs).** The LPR ratios are 2.5 (*black traces*), 5 (*red traces*), and 10 (*blue traces*). *A,* transient currents for wt XylE induced by d-xylose (*left*) and d-glucose (*right*). The current decay of all traces was fitted using the given biexponential function yielding two decay time constants for each trace. For d-xylose, both decay times gradually increase with increasing LPR, which suggests the underlying electrogenic reaction is transport. For d-glucose, the fast decay time is unaffected by LPR, a defining characteristic for a PSS current. The slow decay time varies with LPR but not systematically. The lower decay time value for LPR 10 might be explained by errors during the fitting procedure because of the low current amplitude. Insets show the currents normalized to their peak values. *B,* normalized transient currents for wt LacY using the substrates TDG (*left*) and d-lactose (*right*). The relation between current decay and LPR shows that the measured currents are transport currents for both substrates. *C,* transient currents for wt GlcP. Only PSS currents are detected. Taken from Ref. (37). TDG, β-d-galactopyranosyl-1-thio-β-d-galactopyranoside.

The main substrate of each transporter is marked bold. *K*_*M*_ and *I*_max_ were determined using the Michaelis–Menten equation (Fig. 2, *red curves*). The respective values for the main substrates were taken from Ref. (30). *I*_max_ and *K*_*M*_ are the mean of at least three different measurements. For minor substrates, *I*_max_ is given in percent of *I*_max_ of the main substrate.

a It was shown that wt XylE does not catalyze d-glucose/H^+^ symport (Fig. 3, (6, 31, 32)). Hence, the given half saturation constant should be viewed as an apparent EC_50_, instead of a real *K*_*M*_.

b No concentration dependence was measured for some minor substrates. The given value represents the ratio of the peak current compared with the peak current of the main substrate at the concentrations indicated in Figure 1 rather than *I*_max_.

The peak currents measured at different concentrations of a selection of transported sugars reveal the substrate concentrations at which the uptake proceeds at half-maximal rate, *K*_*M*_. The *K*_*M*_ values for the main substrate sugar and sugars transported less efficiently (minor substrates) by the respective symporter are found in the low millimolar range (Fig. 2 and Table 1). These results compare with *K*_*M*_ values found in transport experiments using radioactively labeled sugars, although the literature values are somewhat lower: XylE (d-xylose: 0.1–0.5 mM) (25, 26), FucP (l-fucose: 20–40 μM) (27, 28), and LacY (lactose: 0.5–3 mM) (9, 29). However, it has been shown for LacY that *K*_*M*_ varies depending on experimental conditions, for example, the energization state of the membrane (9): The apparent *K*_*M*_ for active transport is ∼100-fold lower than the apparent *K*_*M*_ for facilitated diffusion. Therefore, a meaningful comparison of *K*_*M*_ concentrations for different substrates and different transporters is only possible under identical experimental conditions. In SSM-based electrophysiology, membrane potential is usually 0 mV when the peak current is measured. On the other hand, conventional electrophysiology applies voltage, and radioactive uptake experiments mostly involve membrane potential as driving force.

The main substrates for XylE and LacY display higher *K*_*M*−_ and higher *V*_max_ values over those obtained for the minor substrates. In FucP, l-fucose shows the lowest *V*_max_ combined with the lowest *K*_*M*_ concentration (∼7–12-fold over *K*_*M*_ obtained for minor substrates). However, the transport *V*_max_ increases only moderately with the minor substrates (Table 1).

It must be noted that using the Michaelis–Menten equation for several minor substrates with FucP, XylE, and LacY do not result in a perfect fit, showing average *R*^2^ values of 0.955 (Fig. 2, *red equation*). This is in contrast to the main sugar substrates, which produce average *R*^2^ values of 0.989 when the Michaelis–Menten equation is used for the fitting (30). For the minor substrates, the application of two more complex models leads to significant improvements in *R*^2^: (1) Introducing negative cooperativity using the Hill equation improves the average *R*^2^ to 0.992 (Fig. 2, *green equation*). (2) The average *R*^2^ is improved further to 0.998 when a double Michaelis–Menten equation is used (Fig. 2, *blue equation*). Reasons for applying these models are given in the *Discussion* section.

### Differentiation between transport and pre steady-state currents

Another striking observation is that d-glucose induces slow transient currents in wt XylE (Figs. 1 and 2), although uptake assays with radioactively labeled d-glucose and competition-uptake experiments against d-xylose revealed that d-glucose is not transported by XylE, but it binds to the same binding location as d-xylose (6, 31, 32). SSM experiments using proteoliposomes with different lipid-to-protein ratios (LPRs) reveal that the decay time of the d-glucose-induced transient currents does not systematically depend on the protein density, whereas the decay time of d-xylose-induced currents highly depends on the LPR (Fig. 3*A*). For transport currents, the current decay depends on how fast the membrane is charged upon transport: At low LPR (higher protein density), the initial electrogenic transport is high (higher peak current), leading to a faster rise in membrane potential and a faster current decay (lower decay time constant). This general observation has already been discussed for other transporters, for example, LacY (33), NhaA (34), and Clc (35). And it is also observed for all tested substrates that are transported by the respective transporters: d-xylose in XylE (Fig. 3*A*) and also d-lactose and TDG (for which transport has been shown in Ref. (36)) in LacY (Fig. 3*B*).

Since this relation is not observed for d-glucose, the reaction can be attributed to a slow pre steady-state (PSS) reaction. This is compatible with the absence of d-glucose transport in transport assays using radioactively labeled d-glucose (6, 32). Using the same method, we showed that the fast transient current detected with d-glucose in GlcP from *Staphylococcus epidermidis* represents a PSS reaction and is not related to electrogenic transport (Fig. 3*C*, (37)). For PSS reactions, the decay time does not depend on LPR but represents the rate constant of the observed reaction (*τ* = 1/*k*_obs_).

### Sugar-induced PSS charge displacement

Beside the slow transient currents representing transport (or a slow PSS reaction in case of d-glucose-induced currents in XylE), PSS charge displacements in the transport process generate fast transient currents in all transporters (23). In LacY, binding of sugar is associated with a weakly electrogenic PSS charge displacement representing ∼6% of an elementary charge (38), and for XylE, the same effect of similar origin with an even higher amplitude was reported recently (30).

Because the currents generated upon sugar/H^+^ translocation usually overlay the PSS current displacement (30, 33, 38, 39), in wt transporters, the PSS currents are not observed over a wide pH range. However, under conditions where H^+^ translocation does not occur, that is, extreme pH or with inactivated steady-state H^+^-translocation activity (LacY E325A, XylE D27N, and FucP D46N), those PSS reactions can be analyzed individually.

The amplitudes recorded at the same pH for wt and mutant transporters are of the same order of magnitude but vary significantly between the transporters (Table 2). Therefore, in mutant and wt transporters, the same reaction is observed, but the reaction obviously differs between XylE, FucP, and LacY.

**Table 2:** Overview of *K*_*D*_ values, transferred charges (Q), and decay time constants (*τ*) for transport-deficient mutants (“mut”: D27N XylE, D46N FucP, and E325A LacY) and different substrates

Transporter	Substrate	A	B	C	
		*K*_*D*_ (mM) mut	*k*_obs_ (s^−1^) mut	Q (pC)	
				Mut	wt
XylE (D27N XylE)	d-xylose (30 mM)	1 ± 0.1 (pH 7.6)	300 ± 30	6.8 ± 1.5 (pH 7.6)	4.0 ± 1.4 (pH 3.2)
	d-glucose (30 mM)	1.9 ± 0.35 (pH 7.6)	300 ± 40	1.1 ± 0.3 (pH 7.6)	2.5 ± 1 (pH 3.0)
FucP^a^ (D46N FucP)	l-fucose (30 mM)	NA	100 ± 20^a^	−1.2 ± 0.6 (pH 10.5)	∼0.5^b^ (pH 10.5)
LacY (E325A LacY)	d-lactose (100 mM)	20 ± 3 (pH 7.6)	260 ± 60	3.1 ± 1.8 (pH 7.6)	2.6 ± 0.6 (pH 5.0)
	Lactulose (100 mM)	4.6 ± 0.8 (pH 7.6)	380 ± 40	2.2 ± 0.5 (pH 7.6)	NA
	TDG (5 mM)	0.18 ± 0.03 (pH 7.6)	280 ± 20	0.5 ± 0.6 (pH 7.6)	NA

All values are determined from at least three different datasets.

**Figure 4:**
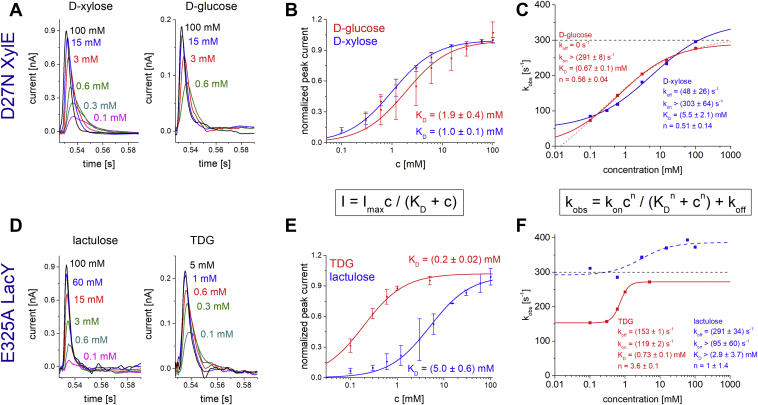
**Substrate dependence and determination of *K***_***D***_**values for different substrates with D27N XylE (*top*) and E325A LacY (*bottom*).** All measurements were performed at pH 7.6 using the high time-resolution set up. The *K*_*D*_ values obtained from fits using the Michaelis–Menten equation, transferred charges (Q), and decay time constants of the transients (*τ*) of all tested substrates are summarized in Table 2. *A,* representative and substrate-dependent transient currents of D27N XylE induced by d-xylose (*left*) and d-glucose (*right*). All traces visualized within the same graph are recorded from the same sensor. *B,* substrate-dependent peak currents induced by d-glucose (*red*) and d-xylose (*blue*). All data points represent normalized and averaged values from n = 3 different sensors. Error bars reflect standard deviations. Before averaging datasets from different sensors, each dataset has been normalized to its *I*_max_ value. The fit equation is given below the graph. *C,* fit of rate constants for sugar binding (*k*_on_) and release (*k*_off_) from the concentration-dependent *k*_obs_ (1/*τ*), which has been obtained by monoexponential fit of the current decay using the equation *I* = *A* ∗ exp(−*t*/*τ*). The fit equation is given below the graph. The *black dashed line* represents the upper limit of the time resolution of the measurements. Values approaching or exceeding this line are not reliable and should be viewed as a lower limit for the real value. The *red dashed line* shows the fit for d-glucose with *R*^2^ = 0.998, which approaches a negative *k*_off_. Hence, the fit for d-glucose was done by setting *k*_off_ = 0 (*red solid line*) also achieving an exceptionally good fit of the dataset (*R*^2^ = 0.997). *D,* representative transient currents of E325A LacY induced by lactulose (*left*) and TDG (*right*). *E,* data points and fits for lactulose- and TDG-induced peak currents obtained with E325A LacY as described in *B*. *F,* fit of rate constants as described in *C*. The fit for lactulose is not reliable since *k*_obs_ is in the range of the time resolution of measurements for all concentrations used. TDG, β-d-galactopyranosyl-1-thio-β-d-galactopyranoside.

**Figure 5:**
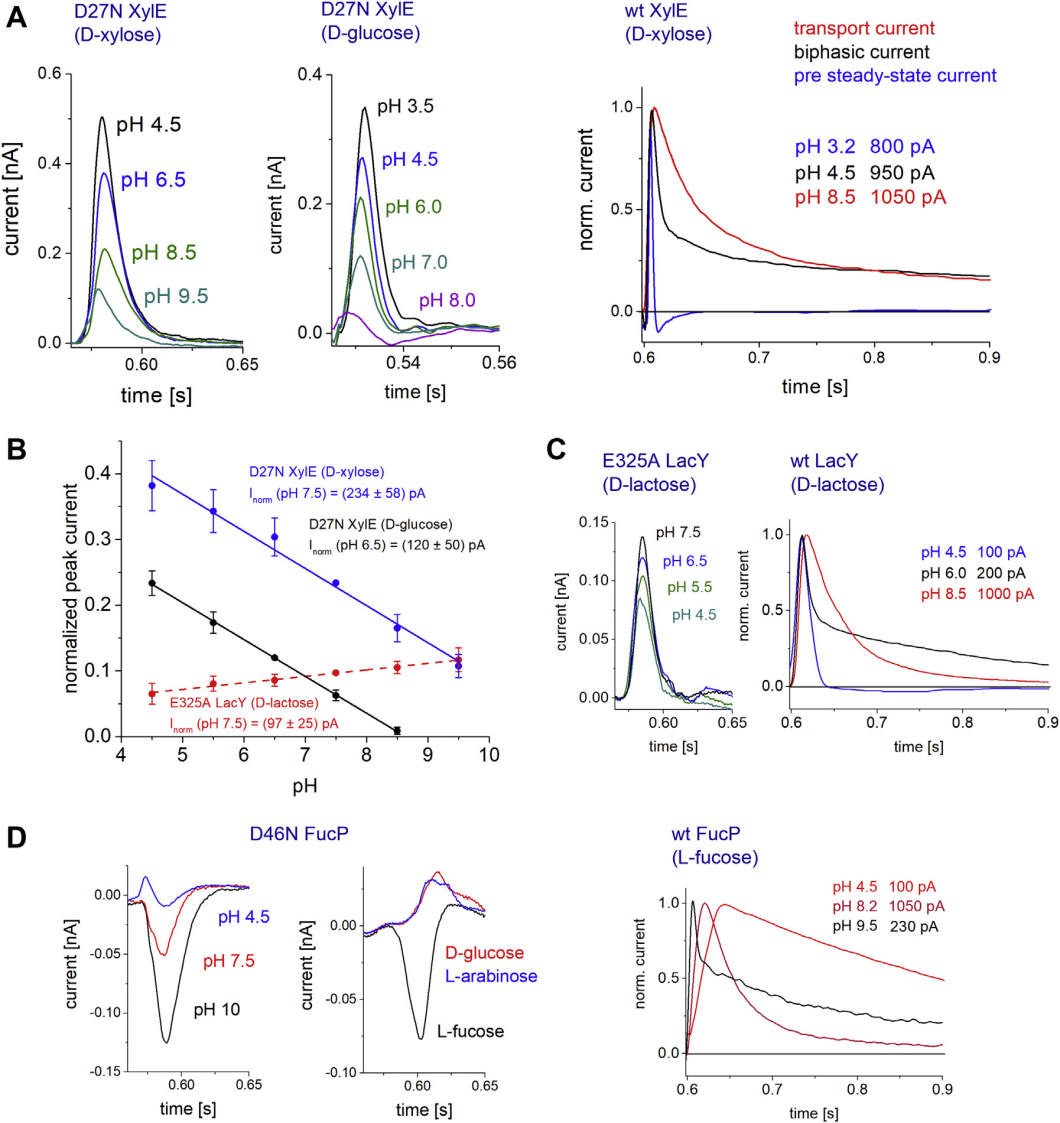
**pH dependence of the PSS currents in mutant and wt transporters.** Substrates and pH values are indicated. All traces visualized within the same graph are recorded from the same sensor. For mutant transporters, only PSS currents are detected. For wt transporters, PSS currents (*blue traces*) or transport currents (*red traces*) dominate the overall current trace at different pH ranges. In between these pH ranges, biphasic currents (*black traces*) are detected, and both PSS and transport currents are identified at the same time. For better visibility of the different signal shapes, all currents for wt transporters have been normalized to their peak current values. The absolute peak currents are indicated in the graph. *A,* pH dependence of transient currents for D27N XylE (*left*) and wt XylE (*right*). For wt XylE, fast PSS currents dominate in the acidic pH range (*blue trace*), whereas a slow decaying transport current dominates in the alkaline pH range (*red trace*). At pH 4.5, a biphasic current is detected (*black trace*). *B,* pH dependence of peak currents for D27N XylE and E325A LacY. All data points represent normalized and averaged values from n = 3 different sensors. Error bars reflect standard deviations. Peak currents obtained from the same sensor (one dataset) were normalized to the peak current at pH 7.5 (for d-xylose-induced currents in D27N XylE and d-lactose-induced currents in E325A LacY) and pH 6.5 (for d-glucose-induced currents in D27N XylE), followed by averaging of datasets. Subsequently, the averaged datasets were again normalized to the mean peak current as indicated (*I*_norm_) to reflect the different current amplitudes across transporters and substrates. *C,* pH dependence of transient currents for E325A LacY (*left*) and wt LacY (*right*). *D,* pH dependence of transient currents for D46N FucP (*left*) and wt FucP (*right*). The PSS current for D46N FucP is dominant at alkaline pH and shows a negative amplitude; it is not observed at acidic pH. This is in agreement with biphasic currents in wt FucP in the alkaline but not in the acidic pH range. When different sugar species are used to activate D46N FucP, only l-fucose induces PSS currents. Nonsubstrate sugars like d-glucose and l-arabinose do not generate PSS currents but small solution exchange artifact currents of positive amplitude. PSS, pre steady-state.

A, *K*_*D*_ values were determined by fitting the peak currents of mutant transporters using a hyperbolic equation without Hill coefficient (Fig. 4). B, To determine the rate constants of the PSS reaction from the transient currents, saturating substrate concentrations were used as indicated within the “substrate” column. First, the decay time constant *τ* was determined using a monoexponential fit (*I* = *A* ∗ exp(−*t*/*τ*)) of the transient current decay followed by conversion to *k*_obs_ v*ia k*_obs_ = 1/*τ*. The decay time constants of the transients induced by all substrates reflect the time resolution of the setup, rendering the reaction *k*_obs_ = 1/*τ* > 300 s^−1^ fast for all tested substrates. For FucP, the low time-resolution set up was used, which explains the somewhat higher decay time constant (lower *k*_obs_) reflecting the lower time resolution of the system. C, To determine the transferred charge (Q) from the transient currents, saturating substrate concentrations were used as indicated within the “substrate” column. Q was determined by integration of the transient current. For comparison, the PSS charge translocated by wt transporters is shown (*right column*). In wt and mutant transporters, different pH values were used as indicated, since PSS currents in wt transporters do overlay with transport currents at physiological pH conditions (Fig. 5, (30)). A similar charge transfer in wt and mutant indicates that equivalent reactions are observed in both transporter variants.

Abbreviation: NA, not applicable.

a All datasets were generated with the high time-resolution set up, except the dataset for FucP, which was recorded using the low time-resolution set up.

b For wt FucP, the PSS current could not be observed isolated from the transport current. Hence, the transferred charge is only a rough estimation.

Titrations of D27N XylE (Fig. 4, *A* and *B*) or E325A LacY (Fig. 4, *D* and *E*) with different sugar concentrations result in hyperbolic-binding characteristics of the respective peak currents. Note that these transient currents represent binding processes rather than transport and are characterized in the following by *K*_*D*_ values (summarized in Table 2). The obtained values for XylE and xylose (1.0 ± 0.1 mM) are similar to the *K*_*D*_ values obtained by isothermal titration calorimetry (0.4 mM) (32), and the LacY titration with lactose yields a *K*_*D*_ of 20 ± 3 mM, which corresponds to values found by protection against labeling of single-Cys LacY (1–10 mM) (40, 41). Also, the *K*_*D*_ values for the minor substrates of XylE and LacY (d-glucose: 1.9 ± 0.4 mM and TDG: 0.18 ± 0.03 mM, respectively) are in good agreement with the values obtained with isothermal titration calorimetry (XylE/d-glucose: 0.77 mM and LacY/TDG: 0.08 mM) (32, 42). Because of the low electrogenicity of the l-fucose–binding reaction to FucP, a *K*_*D*_ value was not determined.

The decay times of the transient currents are derived from monoexponential fits of the current decay and correlate directly with the rate of the recorded PSS reaction (*τ* = 1/*k*_obs_) (Table 2). The decay times for all tested substrates at substrate-saturating conditions with E325A LacY or D27N XylE exhibit only insignificant variance and correlate closely with the resolution limit of the setup (23), rendering it a fast reaction with a rate of >300 s^−1^. Plotting *k*_obs_ against sugar concentrations reveals the rate constants for sugar binding (*k*_on_) and release (*k*_off_) individually using the equation given in Figure 4. This analysis shows that *k*_on_ is limited by the time resolution of the instrument—for all tested substrates (Fig. 4, *C* and *F*). In contrast, *k*_off_ is small enough to be determined. Most interestingly, *k*_on_ is similar for d-glucose and d-xylose binding to XylE (>300 s^−1^), but *k*_off_ is 50 s^−1^ for d-xylose and not distinguishable from 0 s^−1^ for d-glucose, in agreement with d-glucose acting as an inhibitor for d-xylose transport.

Comparisons of the amplitudes or displaced charges induced by binding of the main *versus* minor substrates reveal a striking difference (Table 2): The currents recorded with minor substrates show lower amplitudes and smaller displaced charges compared with the currents recorded with the main substrate of the respective transporter under saturating conditions. This observation indicates that binding of different sugars leads to distinct displacements of charges depending on the identity of the sugar. Thus, the binding of different substrates induces distinct transporter states.

### pH dependence of transport and PSS currents

MFS sugar transporters show a characteristic pH dependence, which can be explained by proton coupling. At extreme acidic and alkaline pH, transport is inhibited, yielding a bell-shaped pH activity curve. At acidic pH, proton release becomes rate limiting, slowing down transport rates, whereas proton depletion at alkaline pH slows down proton binding and the overall transport rate decreases (30).

The pH dependence of the PSS currents cannot be analyzed using wt transporters. The charge translocation resulting from multiple full transport cycles is several orders of magnitude larger and overlays the smaller PSS charge translocations under most pH conditions (Fig. 5). When analyzing the pH dependence of sugar-induced transient currents in wt transporters, both transport and PSS currents appear simultaneously at specific pH conditions (*e.g.*, Fig. 5*A*, wt XylE, *black trace*): This is when transport is slowed down significantly at extreme acidic or alkaline pH values. The exact pH values to observe both reactions differ between XylE, LacY, and FucP and depend not only on the p*K* values for proton release and binding (30) but also on the pH dependence and electrogenicity of the PSS reaction (Fig. 5*B*).

For a complete analysis of the pH dependence of PSS currents, we used the transport-deficient mutants. The pH dependence of the current amplitude reveals a linear characteristic with a positive slope for E325A LacY but a negative slope for the D27N XylE mutant (Fig. 5*B*). The missing hyperbolic titration transition, which is observed for the wt transporters (30), indicates that the signal cannot be attributed to an individual side chain but represents rather a collective charge displacement. Interestingly, pH-dependent d-xylose-induced and d-glucose-induced PSS currents in wt XylE exhibit the same slope; the PSS currents with d-xylose are 165 pA higher than that for d-glucose, independent of pH. The pH dependence of PSS currents of the transport-deficient mutants matches with that of the wt transporters: For wt XylE, the PSS current is larger at acidic pH compared with alkaline pH (Fig. 5*A*), whereas for wt LacY, the PSS currents are larger at alkaline pH compared with acidic pH (Fig. 5*C*, (30)).

Since the rate of the observed PSS reaction is limited by the time resolution of the setup (>300 s^−1^) and faster than the transport rate of LacY (30 s^−1^, (29)), the charge translocation likely represents a reaction directly following sugar binding, for example, the fast movement of charged amino acids, shielding of charged protein residues by the sugar, or tilting of dipolar transmembrane helices. In the following sections therefore, the observed reaction is referred to as *electrogenic binding*.

The PSS charge displacement induced by sugar addition to FucP has been long missed because of its low amplitude (Fig. 5*D*). The binding of l-fucose to wt FucP reveals only a weak electrogenic PSS charge displacement with the same polarity as recorded with XylE and LacY (Fig. 5*C*, (30)). However, the FucP mutant D46N with inactivated H^+^-translocation activity displays pronounced charge displacements, although with the opposite polarity compared with the wt protein or to LacY and XylE mutants (Fig. 5, *A* and *C*). This charge displacement is specific for FucP substrates and absent in traces obtained with other nonsubstrate sugars (Fig. 5*D*, *left*). The peak current in D46N FucP dominates at very alkaline pH and is almost absent at acidic pH (Fig. 5*D*, *left*), in agreement with the appearance of fast transients in wt FucP at alkaline pH (Fig. 5*D*, *right*, (30)).

### d-glucose transport by XylE mutant Q175I/L297F

**Figure 6:**
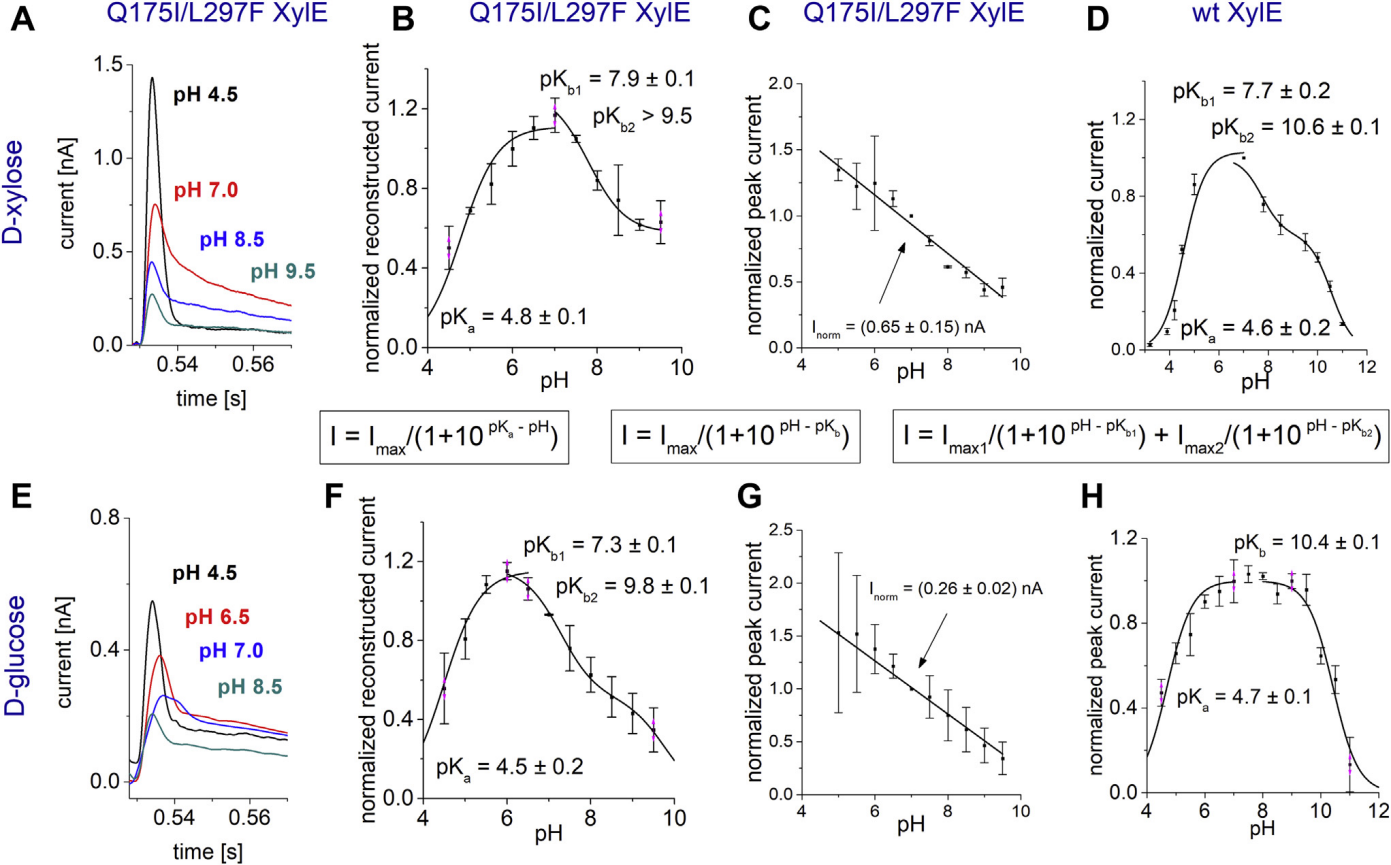
**pH dependence of****d****-xylose (*top*) and****d****-glucose (*bottom*) induced currents in Q175I/L297F XylE.** For comparison, the d-xylose- and d-glucose-induced pH dependence of wt XylE (*D* and *H*) is shown. All data points represent normalized and averaged values from n = 3 different sensors. Error bars reflect standard deviations. Fit equations to derive p*K* values from pH-dependent currents are indicated. *A* and *E,* transient currents for different pH values as indicated. All traces visualized within the same graph are recorded from the same sensor. *B* and *F,* normalized and averaged reconstructed transporter currents for different pH values. Reconstructed currents reflect the steady-state transport rate of the transporter with the respective substrate. The pH dependence is fitted separately using a single titration equation for the acidic pH range and a double titration equation for the alkaline pH range. The p*K* values obtained from the fits are indicated. *C* and *G,* peak currents for different pH values as described for Figure 5*B*. The peak currents are dominated by the fast PSS component of the transient current. Accordingly, it shows a linear pH dependence similar to that observed for D27N XylE (Fig. 5*B*). The normalized peak current *I*_norm_ is indicated. *D* and *H,* pH dependence of peak currents in wt XylE. The plot for d-xylose-induced currents is taken from Ref. (30) for comparison. A double titration equation is required to fit alkaline downregulation of H^+^/d-xylose symport. Q175I/L297F XylE transports both d-glucose and d-xylose and likewise requires two p*K* values to fit alkaline downregulation. In contrast, d-glucose is not transported by wt XylE, and alkaline downregulation of d-glucose-induced currents are described sufficiently using only one p*K* value. This indicates that sugar transport is modulated by two distinct proton-binding sites but not the d-glucose-induced PSS reaction in wt XylE. PSS, pre steady-state.

As mentioned previously, d-glucose binds but is not transported by wt XylE (6, 31, 32). But like d-xylose, d-glucose induces slow peak currents with wt XylE at pH 7.0 exhibiting amplitudes of 26% compared with d-xylose-induced currents, which represent full transport cycles (Fig. 1*A* and Table 3). This d-glucose-induced slow peak current is attributed to a slow PSS reaction, since the rate constant *k*_obs_ does not depend on protein density (Fig. 3*A*), and d-glucose is known to be not transported by wt XylE (6, 31, 32). However, the XylE double mutant Q175I/L297F exhibits significant d-glucose transport (6). For better understanding of the d-glucose-induced slow PSS currents, we examined the pH profile for d-glucose-induced and d-xylose-induced currents as well as the *K*_*M*_ values in wt and Q175I/L297F XylE (Fig. 6 and Table 3).

**Table 3:** Overview of different parameters derived from d-xylose-induced and d-glucose-induced currents with three XylE transporter variants

XylE variant	Substrate	A	B	C		D		
		EC_50_ (mM)	*I*_Peak_ (pA) fast component	*I*_max_ (nA) slow component	Radioactive counterflow activity	p*K*_a_	p*K*_b1_	p*K*_b2_
Q175I/L297F	d-xylose	1.4 ± 0.6	520 ± 90	0.4 ± 0.1 (22%)	75%	4.8 ± 0.1	7.9 ± 0.2	>10
	d-glucose	1.5 ± 0.8	240 ± 40	0.2 ± 0.05 (**11%**)^c^	**10%**^c^	4.6 ± 0.1	7.3 ± 0.1	9.8 ± 0.2
wt	d-xylose	1.7 ± 0.2	<1800	1.8 ± 0.5 (**100%**)^a^	**100%**^a^	4.6 ± 0.2	7.7 ± 0.2	10.6 ± 0.1
	d-glucose	0.4 ± 0.1	<470	0.47 ± 0.1 (**26%**)^b^	**<1%**^b^	4.7 ± 0.1	—	10.4 ± 0.1
D27N XylE	d-xylose	1 ± 0.1	500 ± 100	<0.05	90% (<1% in uptake assay)	—	—	—
	d-glucose	1.9 ± 0.35	60 ± 25	<0.05		—	—	—

All parameters were taken from datasets generated at pH 7.6 and under saturating substrate concentrations. A, EC_50_ refers either to the *K*_*M*_ value for transport of the respective substrate when the slow component was used for analysis (Q175I/L297F XylE) or to the *K*_*D*_ or apparent affinity of the respective substrate when the fast component is analyzed (D27N XylE). In wt XylE, the EC_50_ for d-xylose equals *K*_*M*_, whereas the EC_50_ for d-glucose represents neither transport activity (*K*_*M*_, Fig. 3) nor electrogenic binding (*K*_*D*_, a fast d-glucose binding-related PSS current is still detected in D27N XylE, Fig. 5*A*). It should be rather viewed as an apparent EC_50_ for the underlying reaction as discussed in the main text. B, *I*_Peak_ refers to the peak current of the fast component, which at pH 7.6 is only observed for D27N XylE and Q175I/L297F XylE. In wt XylE, the transport current defines the peak current. Hence, the fast current peak value is lower than the transport current that is given as an upper limit. C, *I*_max_ was derived from the hyperbolic fit of the substrate dependence for the slow component in Q175I/L297F XylE (reconstructed transporter current) and for the peak current in wt XylE; both values are dominated by electrogenic transport activity. D, p*K* values were derived from pH-dependent peak currents (wt XylE) or reconstructed transporter currents (Q175I/L297F XylE) using single (acidic pH range, p*K*_a_) or double (alkaline pH range, p*K*_b1_ and p*K*_b2_) titration equations (Fig. 6). The p*K* values for d-xylose-induced currents in wt XylE were taken from Ref. (30). In wt XylE, the pH dependence for d-glucose-induced currents can be described by a single p*K*, whereas transport of d-xylose in wt XylE and transport of d-xylose and d-glucose in the double mutant requires two p*K*s to describe alkaline downregulation. Therefore, an unknown component with p*K* ∼7.5 is modulating transport but not the d-glucose-induced PSS reaction in wt XylE.

a For better comparison, the transport activity is also given as normalized value in relation to d-xylose transport in wt XylE (100%). The right column illustrates the relative counterflow activity using radiolabeled substrates (data taken from Ref. (6)).

b As described in the main text, the d-glucose transport activity in the counterflow assay does not fit with the relative peak current in the SSM experiment—another indicator that the d-glucose-induced current in wt XylE is not related to transport.

c On the other hand, the d-glucose-induced relative current in Q175I/L297F XylE fits very well with the transport activity in the counterflow assay, indicating that the slow component of the double mutant represents transport.

SSM recordings on Q175I/L297F XylE using d-glucose and d-xylose reveal transient currents with biphasic shape (Fig. 6, *A* and *E*). The slow component represents the proton-coupled sugar influx, although showing a markedly lowered *V*_max_ compared with d-xylose transport in wt XylE. The relative *V*_max_ for d-glucose transport in the double mutant compared with d-xylose transport in wt XylE agrees with the relative transport activities measured in radioactive counterflow assays, which is about 10% (Table 3). This indicates that d-glucose-induced currents observed in Q175I/L297F XylE reflect d-glucose transport. In contrast, the d-glucose currents observed in wt XylE do not reflect d-glucose transport: the relative current amplitudes for d-glucose compared with d-xylose (26%) do not match with the relative transport activities in counterflow experiments (<1%) (Table 3).

The double mutant shows similar properties for d-glucose-induced currents like wt XylE for d-xylose-induced currents: (1) The pH profile of the reconstructed transport-related current component for d-xylose and d-glucose (Fig. 6, *B* and *F*) reveals similar p*K* values compared with the d-xylose-induced pH profile of wt XylE (Fig. 6*D*). (2) The fast component representing electrogenic binding shows linear pH dependence (Fig. 6, *C* and *G*), similar to the peak currents observed with D27N XylE (Fig. 5*B*). In contrast to wt XylE, D27N XylE exhibits the fast electrogenic binding signal at all pH values, because the steady-state sugar/H^+^ transport is abolished in D27N XylE, but the charge translocation upon sugar binding shows similar properties as in the wt. (3) The *K*_*M*_ for d-glucose-induced peak currents in the double mutant also verges on the d-xylose-induced peak currents in wt (Table 3).

In contrast to the similar properties for d-glucose-induced and d-xylose-induced currents in the double mutant and d-xylose-induced currents in wt XylE, which all relate to steady-state transport, the properties of d-glucose-induced currents in wt XylE show significant differences for three reasons (Table 3): (1) The alkaline downregulation of d-xylose transport in wt XylE and d-glucose as well as d-xylose transport in the double mutant are all described by two distinct p*K* values (p*K*_b1_ ∼ 7.5 and p*K*_b2_ > 9.5) (Table 3 and Fig. 6, *B*, *D*, and *F*). In contrast, the downregulation of the d-glucose-induced reaction at alkaline pH is described by one single p*K*_b_ (10.4), rendering the so far unknown component with p*K*_b1_ ∼ 7.5 as a modulator for steady-state transport activity but not for the d-glucose-induced PSS reaction in wt XylE. (2) d-glucose-induced fast charge translocation in D27N XylE (60 pA) is much lower compared with the reactions induced by d-xylose in D27N XylE (500 pA) as well as d-glucose in the double mutant (240 pA) (Table 3). This suggests that the transporter state of wt XylE after d-glucose binding (no sugar translocation observed in radioactive counterflow) differs from the state of D27N XylE after d-xylose binding; and it differs from the states of the double mutant after binding of d-xylose or d-glucose (sugar translocation observed in radioactive counterflow). (3) The *K*_*M*_ for d-xylose in wt and d-xylose and d-glucose in the double mutant is identical within standard deviation (∼1.5 mM), whereas d-glucose shows a much higher affinity to wt XylE (0.4 mM) (Table 3), possibly hindering sugar release.

### Analysis of the side-chain interaction network

Since the major and minor substrates induce different PSS charge displacements under saturating conditions in the respective transporters (Fig. 1 and Table 3), it follows that the charge rearrangements differ indicating different transporter conformations after electrogenic binding of different substrates. The analysis of the X-ray structures of the apotransporter and in complex with different sugars, by means of the graph theory, revealed that the amino acid interactions within the transporter are influenced by the bound sugar species. Two indicators have been considered in detail: The betweenness centrality (BC), as an indicator of the importance of a specific residue in the network, and the *weighted degree*, as a measure of the residue's interactions (connections to the adjacent residues) weighted by the interconnections intensity of the adjacent residues to other residues.

**Figure 7:**
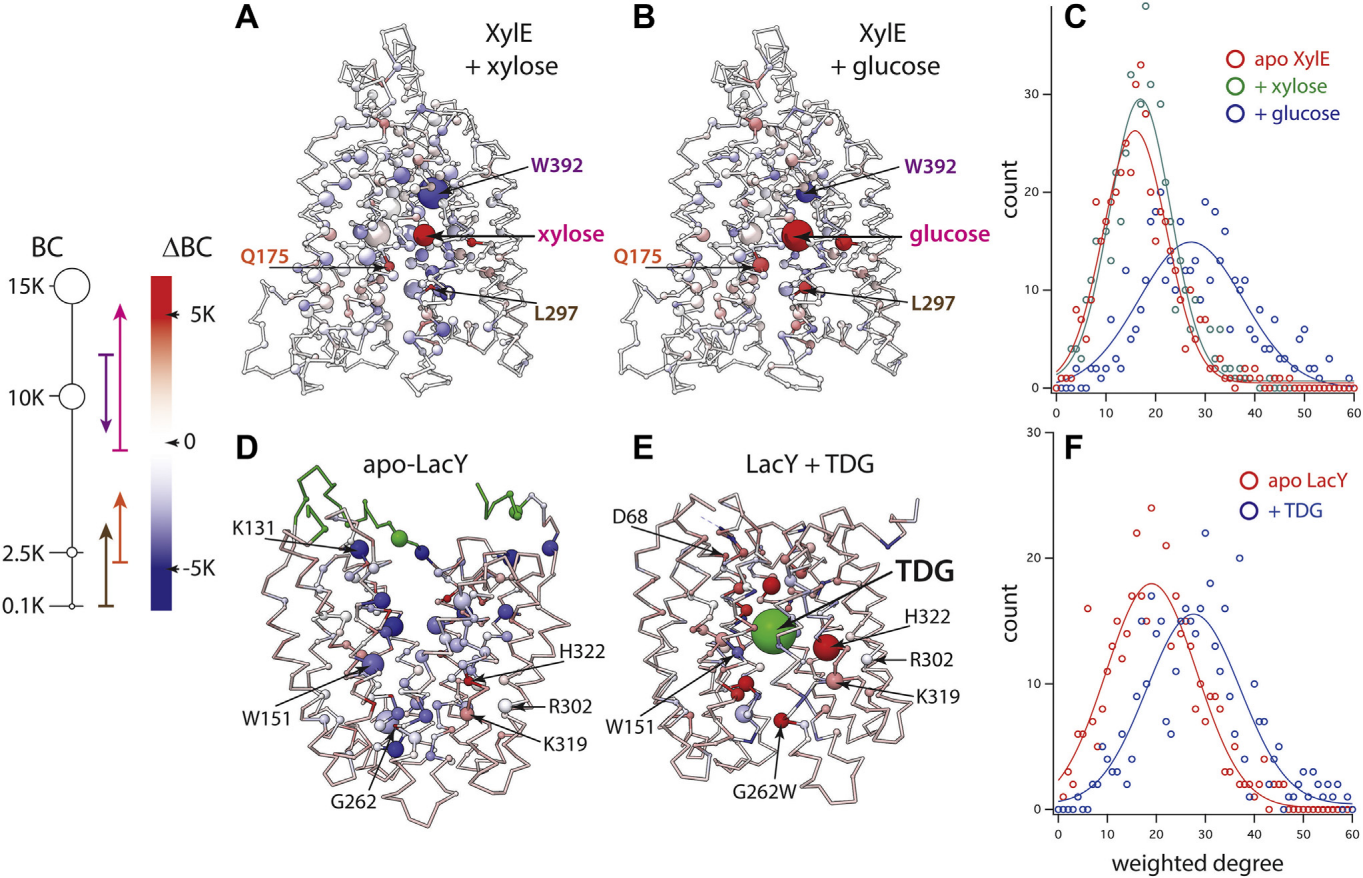
**Fine structure differences of residue interactions in XylE and LacY X-ray structures.***Left panel,* size and color legend for *A* and *B* as well as *D* and *E* (K = 1000). The *arrows* indicate relative changes for the residues Q175 (*orange*), L279 (*brown*), W392 (*violet*), and xylose or glucose (*magenta*). The base corresponds to the respective values in the xylose-bound structure, and the arrow is matched at values in the glucose-bound structure. *A,* Cα trace model of XylE with bound xylose. *B,* XylE with bound glucose. Cα atom size corresponds to the betweenness centrality (BC) of the respective position. The model is colored according to the BC difference of the respective position in xylose-bound XylE and XylE with bound glucose (see legend in the *left panel*). Xylose and glucose are colored according to the BC difference of the two positions; some prominent residues are indicated for orientation. *C* and *F,* weighted degree distribution reported against their occurrence (count). *C,* weighted degree distribution calculated for residues in X-ray structures showing XylE with bound major substrate (Protein Data Bank [PDB] ID: 4GBY, xylose: *green*) or with bound minor substrate (PDB ID: 4GBZ, glucose: *blue*). The *red trace* indicates the distribution found in XylE structure without bound substrate. *D,* Cα trace model of apo-LacY without bound substrate. *E,* Cα trace model of LacY in complex with TDG. The model is colored according to the BC difference of the respective position in apo-LacY and LacY with bound TDG (see legend in the *left panel*). Positions unique to a specific model are indicated in *green* color. *F,* weighted degree distribution calculated for residues in LacY X-ray structures with bound TDG (PDB ID: 4OAA, *blue*) and without bound substrate (PDB ID: 2V8N, *red*). TDG, β-d-galactopyranosyl-1-thio-β-d-galactopyranoside.

By means of the interaction network, the X-ray structures of apparently similar conformations vary significantly with different sugars bound to the transporter as observed in the crystallographic coordinates of XylE with bound d-xylose or d-glucose (Fig. 7, *upper panel*). Remarkable differences are observed for BC values of prominent elements—for example, W392, unlike in the structure model of XylE with bound d-xylose, the value of d-glucose dominates the interaction network in the respective structure (Fig. 7*B*). More importantly, BC values of specific residues shift significantly (Fig. 7: *arrows* in the legend indicate BC shifts for XylE with d-xylose and d-glucose) indicating changes in the organization of the interaction network. Furthermore, the weighted degree distribution, which follows a Gaussian distribution, reveals that the residues in X-ray structures of the transporters average at mean values ranging between 10 and 20 for the apo-bound and main substrate-bound transporters regardless of the resolution limit of the X-ray data (Fig. 7*C*, *red and green*). However, in structures showing the transporter with bound minor substrate, the weighted degree distribution is markedly shifted to higher values (Fig. 7*C*, *blue*).

The graph representing the crystallographic structure of the inward-facing conformation of LacY shows positions with high BC (Fig. 7, *lower panel*), clustering around the central cavity with the exception of few residues of otherwise critical importance, for example, R302 or K319. Binding of TDG, a galactopyranoside that is transported rather slow (*V*_max_ = 21% compared with d-lactose, Table 1), induces large global conformational changes concluding in the alternating-access mechanism (5). The X-ray structure of the substrate-bound LacY shows that the presence of sugar in the binding site causes also significant local changes (Fig. 7*E*) resulting in large shifts in the BC values, a measure of the nodes “importance,” of respective positions. Similar to XylE, the structure showing the transporter with bound minor substrate, TDG, is characterized by a shift of the weighted degree distribution to markedly higher values (Fig. 7*F*, *blue*).

Taken together, the analysis of interaction networks calculated from the available crystallographic coordinates reveals significant differences of the fine structure in different conformers as well as differences in seemingly identical conformations of the transporters bound to different sugars. Both transporters seem to cringe to bound minor substrate in the translocation site.

## Discussion

Here, we focused on XylE as a model for H^+^/sugar symport to investigate electrogenic reactions involved in sugar translocation. In addition, different aspects of the MFS symport mechanism have been resolved also for the related transporters LacY and FucP. This allows for more comprehensive conclusions regarding conformational mechanics associated with the substrate translocation, which are discussed in the following.

### Minor substrates show a Hill-like dependence

The transient currents recorded at different concentrations of some minor substrates (glucose in XylE, arabinose in FucP, and lactulose in LacY) and the respective symporter yield a concentration dependence (Fig. 2), which is not represented by a standard Michaelis–Menten equation, showing average *R*^2^ values of 0.955. This is not the case for the main substrates d-xylose for XylE, l-fucose for FucP (30), and d-lactose for LacY (33). Two different models have been applied to optimize the fit.

The double Michaelis–Menten equation assuming two electrogenic reactions with different apparent affinities models our data best, indicated by the highest *R*^2^. This would be the case when both PSS currents and transport currents affect the peak current amplitude showing similar *I*_max_ values. In contrast to major substrates, the ratio between transport and PSS currents in wt transporters might be decreased, leading to biphasic concentration dependences. However, we determined *K*_*M*_ values using the low time-resolution setup to reduce possible impacts of fast PSS currents on the peak current amplitude (Fig. 2), and PSS currents in transport-deficient mutants are low in amplitude compared with transport currents in wt transporters. Nonetheless, we have checked for this possibility by using an alternative analysis procedure, based on the following assumptions: PSS currents only affect the peak current amplitude because of their fast charge displacement, whereas transport should dominate the overall charge displacement when a longer period is observed. Hence, the current integrals should be less affected by the low PSS charge displacements. When the PSS current is the reason for the biphasic concentration dependence of the peak currents, the fit is expected to be monophasic when the peak integrals are used instead. When we used integrals for the fits, no change of the biphasicity was observed (data not shown), indicating that the PSS reaction does not affect the determined concentration dependence for minor substrates and that electrogenic transport dominates the peak current in wt transporters.

Similar *R*^2^ values are obtained when a Hill model with a Hill coefficient <1 is applied. This is typically interpreted as negative cooperativity. In most cases, cooperativity is associated with multimeric enzymes possessing multiple interacting ligand-binding sites. But also in monomeric enzymes with single binding sites, cooperativity is a well-documented phenomenon (43). Positive (44) as well as negative cooperativity (45) has been observed for instance in sugar kinases, and different kinetic models have been proposed to account for this behavior. With regard to our sugar transporters, the “random ternary complex mechanism” seems to be most relevant. It was suggested to explain cooperativity observed with liver hexokinase (44), an enzyme catalyzing the phosphorylation of glucose by MgATP. It is a bireactant enzyme with two substrates (glucose and MgATP), a situation analogous to sugar/H^+^ cotransporters (two substrates, H^+^ and sugar). It seems conceivable that the “random ternary complex mechanism” is also operative in sugar/H^+^ cotransporters and that random binding of H^+^ and sugar is responsible for the negative cooperativity observed with certain substrates. Since the type and extent of cooperativity critically depends on the involved rate constants (46), certain substrates may show cooperativity, whereas others do not, for example, the main substrates (d-xylose in XylE, l-fucose in FucP, and d-lactose in LacY) (30).

### C4 of the sugar is essential for transport

In striking similarity to LacY (12, 24), all examined MFS sugar/H^+^ symporters rely on the orientation of the OH group at position C4 for transport (Fig. 1). When exchanging the residues at position C5 or C6 of the sugar, the transport rate is only affected slightly, while changing the orientation of the C4 OH group compared with the main substrate (l-idose in XylE and d-altrose in FucP) leads to a significant decrease in the observed transport rate (Fig. 1). In the structures of XylE (32) and LacY (12, 13) solved with a sugar bound in the substrate-binding site, the C4 OH group of the respective sugar makes close contact to the residue Q175 coordinating a water molecule (30) in XylE and its complements E269 and N272 in LacY (6) making them likely candidates for a primary role in specificity (12). The exchange of the corresponding side chain in FucP, Asn162–>Ala, results in mutants with drastically reduced transport activity (47), indicating that the specificity determinant may be conserved in MFS sugar/H^+^ symporters.

### The PSS signal on the SSM is a monitor for sugar binding

**Figure 8:**
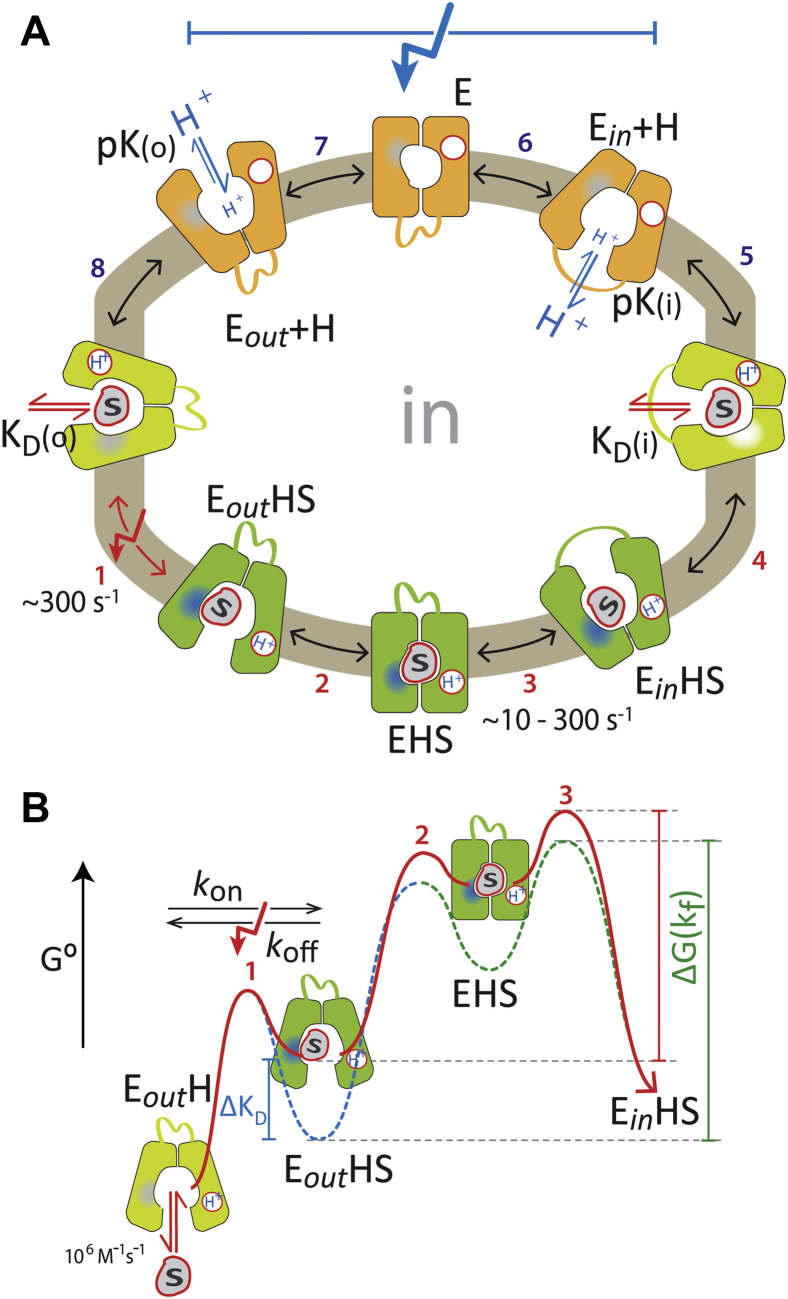
**Overview of the postulated steps in the transport model of MFS sugar/H**^**+**^**symporters.***A,* complete transport cycle. Inward-facing (*right*) and outward-facing (*left*) conformations are separated by the apointermediate conformational cluster (*orange*) and the occluded-intermediate conformational cluster (*green*). Steps are numbered consecutively. The transport cycle is triggered by binding of the sugar (S) to the protonated carrier in the outward-facing conformation (E_out_H). (*1*) Sugar binding induces reorientation of the sugar-binding pocket; this is postulated to be the electrogenic sugar binding, which is measured as a fast PSS current on the SSM, indicated by a *red arrow*; (*2*) induced fit to the occluded intermediate, which is assumed to be the rate-limiting step for H^+^/sugar translocation at physiological pH conditions, except for d-lactose transport in LacY (30); (*3*) opening of the cytoplasmic cavity; (*4*) release of sugar in the inward-facing conformation; (*5*) deprotonation; (*6*) formation of the deprotonated apointermediate; (*7*) conformational transition to the outward-facing conformation and reprotonation of LacY; steps *6* and *7* account for net charge translocation during the transport cycle—indicated by a *blue arrow*—translocating the negatively charged proton-binding site across the membrane; (*8*) sugar binding to the outward-facing protonated carrier, which starts another transport cycle. All steps are reversible (indicated by *double-headed arrows*). *B,* energy diagram for the sugar translocation pathway (steps *1*–*3* in *A*). Substrate (S) binds (*K*_*D*_(o)) to the protonated and outward-facing conformer (E_out_H) inducing a charge rearrangement (*blue patch*). The rate constant for this electrogenic reaction is *k*_obs_ >300 s^−1^ for all tested sugars and has been determined by SSM-based electrophysiology (see main text). The substrate transporter complex (E_*out*_HS) then undergoes a major conformational transition to form the occluded state (*green patch*). This reaction is postulated to be the rate-limiting step within the sugar translocation pathway (with exception of d-lactose transport in LacY), since minor substrates are transported with <30 s^−1^ (see main text). MFS, major facilitator superfamily; PSS, pre steady-state; SSM, solid-supported membrane.

Since the proton-binding site is negatively charged and neutralized in the proton-bound transporter state, we conclude that the predominant electrogenic event in sugar/H^+^ symport is the translocation of the negatively charged H^+^-binding site of the deprotonated and empty carrier (Fig. 8*A*, *steps 6 and 7*). In contrast, mutants defective in sugar/H^+^ symport, which bind ligand normally, exhibit a faster and smaller charge displacement on addition of substrates (Table 2) compared with wt signals at optimal pH (30). Unlike the major electrogenic event of wt transporters, the linear variation of signals with ambient pH observed for the transport-deficient mutants (Fig. 5*B*) indicates that an individual H^+^ acceptor with a defined p*K* is unlikely to be the origin of the charge displacement—rather, the concerted charge rearrangements within the transporter cause the effect. These charge rearrangements can increase (LacY) or decrease (XylE) with pH (Fig. 5*B*) and can also show negative amplitudes (FucP, Fig. 5*D*), which shows that the rearrangements differ between transporters while the process itself is conserved.

It has to be noted that similar PSS currents have been detected in the glucose transporter GlcP from *S. epidermidis* (37) and the melibiose/Na^+^ transporter MelB (48, 49). Recently, we found similar PSS currents in the human d-glucose/Na^+^ transporter SGLT1 (50), which belongs to the solute sodium symporter family. We propose that the underlying electrogenic process of sugar binding may be conserved across all MFS sugar transporters and potentially even beyond.

Because the quantities of charge displacements and decay times induced by sugar binding are similar in the wt and mutant of the same transporter (Table 2), it is evident that similar structural rearrangements are observed in the wt and mutant proteins. The PSS reaction is observed for all transporters as well as all substrates, which makes the PSS signal a good monitor for sugar binding. Interestingly, the major and minor substrates induce different PSS charge displacements (under saturating conditions) in the respective proteins (Table 2). It is therefore obvious that the charge rearrangements are different indicating differences of the conformations after electrogenic binding of different substrates. It is likely that the charge displacement induced by the main substrate represents a conformational transition into an energetically optimal transporter state, favoring the following translocation of the substrate.

### Substrate binding is a multiple step process

With SSM-based electrophysiology, we found an electrogenic reaction following sugar binding. The signal represents a fast reaction for all tested substrates (*k*_obs_ > 300 s^−1^, limited by the time resolution of the instrument). At the same time, sugar substrates other than the main substrates (d-xylose for XylE, l-fucose for FucP, and d-lactose for LacY) are transported at lower *V*_max_ (Table 1), rendering one step during sugar translocation as the rate-limiting slowest step in the transport cycle for minor substrates. Since the transport rate for lactose transport by LacY is only 30 to 50 s^−1^ (29, 33), this rate-limiting step within the sugar translocation pathway must be slower than 30 s^−1^. This is also true for XylE and FucP, since they show similar peak current amplitudes on the SSM, indicating that the transport rates for the main substrates are similar to the transport rate observed for LacY.

Based on these findings, we deduce more than one reaction in the sugar translocation pathway: One electrogenic reaction with a rate constant of *k*_obs_ > 300 s^−1^ for all tested sugars and one rate-limiting reaction with a rate constant of <30 s^−1^ for minor substrates. We postulate that the transport rate-limiting step for the minor substrates (Fig. 8, *green*) is the formation of an occluded state.

For LacY, the alternating-access mechanism and the associated conformational transitions are postulated to involve an induced-fit mechanism caused by the binding of galactosides (3, 12, 19). This implies that the substrate is not optimally liganded in conformations with an open cavity, and the binding of substrate induces a major reorganization in the binding site, leading to formation of an intermediate (the occluded state, (12, 13, 32)) with optimum fit involving side chains from the N-terminal and C-terminal domains. In this mechanism, the increased substrate–protein interaction provides an intrinsic binding energy to lower the energy barrier between the inward-facing and outward-facing conformation of the transporter (3, 51). In other words, the catalytic energy necessary for transport is thought to be recruited from the substrate–protein interaction. In this view, the formation of the occluded state is rate limiting for the sugar translocation pathway, and the occluded state resembles a metastable high-energy occluded intermediate (substrate-bound and protonated) (Fig. 8, EHS) (6).

Kinetic data are available for LacY supporting this conjecture. The binding of *p*-nitrophenyl-d-galactopyranoside to C154G/V331C LacY, a fluorescent-labeled protein in detergent micelles (52), was observed directly with stopped flow experiments. The sugar induced a two-step reaction with rate constants of 10^6^ M^−1^ s^−1^ for the binding reaction and 250 s^−1^ for the following conformational changes (33, 52). The second reaction has a similar rate constant to that observed on the SSM (>300 s^−1^, also shown in Ref. (38)).

Taken together, we postulate three distinct reactions induced by the substrate: (1) fast binding (10^6^ M^−1^ s^−1^) (52); (2) electrogenic rearrangements within the binding site (>300 s^−1^ for all substrate species), possibly also observed in *p*-nitrophenyl-d-galactopyranoside–binding experiments (52); and (3) the rate-limiting formation of the occluded state with rate constants depending on the substrate species and <30 s^−1^ for minor substrates (29).

### Different substrates induce different fine structures of the transporter

Crystallographic structures of XylE and LacY as well as other MFS transporters suggest that the conformational transitions involved in the alternating-access mechanism occur as rigid-body movements of the N-terminal and C-terminal subdomains (12, 53), according to the “rocker-switch” model in which the domains rotate against each other around the middle of the protein (54, 55). However, various biochemical and biophysical approaches provide converging evidence that LacY is highly dynamic (56, 57, 58, 59, 60) and that it undergoes complex conformational changes, which involve essential structural intermediates with distinct energetic, kinetic, and mechanical properties (12, 19, 61, 62).

The inspection of the crystallographic coordinates obtained with XylE or LacY in different conformation reveals significant differences (12, 17, 32). The differences are quantified in terms of the graph theory (63). A protein's crystallographic structure can be represented by a graph of the amino acid interaction network (Fig. 7). The interaction network sufficiently describes the proteins 3D structure for the analysis of conformational changes. However, other than 3D representation of the structure model, the graph representation evaluates the statistical significance of relations between elements of the structure rather than the individual chemistry of particular interactions.

The TDG-bound structure of LacY shows more “intense” interactions (Fig. 7*F*) compared with the apo-LacY structure. Similarly, structures of apo-bound and xylose-bound XylE show a more relaxed network with a lower average interaction degree compared with the glucose-bound XylE (Fig. 7*C*). More interactions likely confine the conformational space of the residues because more interconnections are affected in conformational transitions. Accordingly, the weighted degree distributions shifted to higher values indicate that conformational intermediates on the translocation pathway may be separated by higher activation energy barriers or not accessible at all for the transporter—transport may occur on suboptimal reaction pathways leading to slower rates. Also, different BC values of specific residues are observed with different substrates (Fig. 7, *A* and *B*), implying that the residues are differently involved in the interaction—in other words, d-glucose induces an occluded state with the wrong side chains of XylE.

The induced fit predicts for a transporter that the substrate is not optimally liganded in the ground state similar to enzymes. However, as opposed to enzymes, the chemically unchanged substrate induces a major reorganization in the binding site, leading to formation of an intermediate with optimum fit (the occluded state). Taken together with the results obtained with the SSM-based approach, these findings lead to the conclusion that minor substrates induce a suboptimal conformation for transport, which might be reflected by the smaller charge displacements recorded for minor sugar substrates upon sugar binding.

### Energy profile of sugar translocation and the correlation between *K*_*D*_ and *V*_max_

The hypothetical energy profile for the transport cycle (19) provides a closer view to the reactions in the sugar translocating pathway: The inward-facing and the outward-facing conformers (Fig. 8: E_*in*_H and E_*out*_H, respectively) are separated by a metastable and high-energy occluded intermediate (Fig. 8: EHS). Substrate is thought to stabilize the occluded state and hence lowering the energy barrier between the outward-facing and inward-facing conformations of the protonated carrier. The energy barrier for the protonated carrier (EH) in the absence of sugar must be high to prevent proton leakage.

Figure 8*B* visualizes the postulated steps in the sugar translocation pathway of MFS sugar/H^+^ symporters. The rate-limiting step is the formation of the occluded state, whereas the electrogenic binding defines the determined *K*_*D*_ values (Fig. 8*B*, *blue curve*). We tested the *K*_*M*_ and transport rates of three substrates for each wt transporter. The *K*_*D*_ for different sugar substrates is in tested cases proportional to the *K*_*M*_ (as observed for LacY, Tables 1 and 2). When assuming *K*_*M*_ is proportional to *K*_*D*_, our experimental results show that for the examined transporters, higher affinity correlates with a lower transport rate for the respective substrate. This indeed is an indication that the rate-limiting step directly follows the electrogenic binding. A lower energy of the substrate-bound carrier (higher affinity) correlates with a higher activation energy of the following rate-limiting formation of the occluded state and therefore a lower transport rate (Fig. 8*B*).

Note that in FucP, the main substrate l-fucose has the highest affinity and lowest transport rate, whereas in XylE and LacY, the main substrates d-xylose and d-lactose have the lowest affinity and highest transport rate (Table 1). FucP transport of the main substrate, l-fucose, is achieved by adjusting *K*_*M*_ at a lower level (10 times higher apparent affinity) without significantly reducing *V*_max_ compared with the minor substrates. But in XylE or LacY, the main substrates achieve higher translocation rates in comparison to minor substrates, while displaying lower stability of the transporter/sugar complex (high *K*_*M*_).

The influence of the sugar species on *V*_max_ and the earlier mentioned relations of sugar-induced reactions were analyzed at pH 7.6. This indicates that at physiological pH, the formation of the occluded state is rate limiting for sugar-induced transport as observed on the SSM in most cases. This is compatible with the observation that in XylE and FucP, the transport rate reaches its maximum in the physiological pH range and is therefore not dependent on proton release or binding (30). d-lactose transport in LacY is an exception: Here at pH 7.6, proton release or the rate of the following conformational transition of the empty carrier becomes rate limiting because of the alkaline-shifted pH optimum (30). Since the minor substrates for LacY reduce the transport rate compared with d-lactose, for these substrates also, the formation of the occluded state becomes rate limiting. d-lactose transport in LacY is a special case in this regard: At physiological pH conditions, sugar occlusion is rate limiting for sugar/H^+^ translocation for all wt transporters and tested sugars, except for d-lactose/H^+^ transport in LacY.

It is important to mention that the correlation between *K*_*D*_ and *V*_max_ only works qualitatively—meaning that for the different sugars, the *V*_max_ does not change as much as the *K*_*D*_ change would suggest. The energy profile implies that the change of rate constants of the rate-limiting reaction (∼*V*_max_) should equal the change of *K*_*D*_. If this model is applied, then also the energy level of the transition state is affected, giving a separate influence on the energy profile for different sugars (Fig. 8*B*, *green curve*).

Taken together, the minor substrate species would influence the transport rate by either one or a combination of the following mechanisms: (1) lowering the energy level of the outward-facing conformer (E_*out*_HS) resulting in higher activation energy barrier to the occluded state (EHS) as observed as lower *K*_*D*_ values for the minor substrates in XylE or LacY (Fig. 8*B*, *blue curve*), or (2) by lowering the energy level of the occluded state (EHS) increasing the activation barrier to relax into the open conformers, or (3) by acting on the activation barrier itself (Fig. 8*B*, *green curve*) by restricting the access to optimal reaction pathways by limiting the conformational space. In either way, the transport rate of the minor substrates would be markedly affected. MFS transporters achieve high catalytic efficiency by adjusting the affinity for the substrate and conformational flexibility to maximize translocation rates. As described previously, the correlation between *K*_*D*_ and *V*_max_ shows that *V*_max_ is less affected as the *K*_*D*_ change would suggest. This supports the idea that in addition, the activation barrier is reduced for substrates with lower affinity (Fig. 8*B*, *green curve*).

We conclude therefore that MFS transporters operate in a fashion analogous to enzymes with the exception that the substrate induces transition states of the protein rather than the protein induces transition states of the substrate (64). By this means, the occluded conformation is induced by sugar binding, and binding leads to lowering of the activation energy barriers for the transition between the inward-facing and outward-facing protonated carriers. The observation that different substrates induce different PSS charge displacements indicates divergent transport intermediates with different substrates.

### d-glucose acts as an inhibitor for XylE by interactions with Q175 and L297

It was shown that d-glucose binds to but is not transported by wt XylE (6, 31, 32), which is supported by our measurements using different LPRs for XylE on the SSM (Fig. 3). d-glucose acts as an inhibitor for XylE, which is beneficial for the organism: As soon as d-glucose is available, d-xylose uptake is not required for energy metabolism. In fact, the additional enzymes XylA and XylB have to be expressed to convert d-xylose into the common metabolic intermediate xylulose 5-phosphate (65), whereas d-glucose can enter energy metabolism directly.

The existence of the fast electrogenic binding current for d-glucose (*k*_obs_ > 300 s^−1^) shows that the initial electrogenic binding of the sugar follows a similar mechanism like the binding of d-xylose (Fig. 5*A*). In addition, with wt XylE, using the SSM setup, we observe that d-glucose induces slow transient currents with p*K*_a_ and p*K*_b_ values similar to p*K* values measured with d-xylose (Table 3). Still, there is one major difference: alkaline downregulation of d-xylose transport shows two p*K*_b_ values, whereas for d-glucose-induced currents, only one p*K*_b_ value is observed. Since only d-xylose and not d-glucose is translocated by wt XylE (6, 31, 32), this shows the importance of a so far unknown component with the p*K* of 7.7 for transport but not for d-glucose binding.

The existence of a bell-shaped pH dependence requires proton binding (p*K*_o_) and release (p*K*_i_) (30). The d-glucose-induced current in wt XylE therefore can tentatively be assigned to a d-glucose binding induced single turnover proton leakage. d-glucose binding to the protonated carrier in the outward-facing conformation is followed by proton release in an inward-facing conformation. Then the transport cycle stops in a d-glucose-bound conformation, which explains the lack of d-glucose transport observed in radioactive uptake experiments (6, 31, 32) and the observed LPR dependence of the slow PSS current (Fig. 3) at the same time.

In Q175I/L297F, a transport rate of d-glucose of about 10% of that of d-xylose in wt XylE was shown in radioactive counterflow assay (6). In line with this result, the d-glucose-induced slow current component measured with SSM-based electrophysiology using Q175I/L297F XylE is about 10% of that compared with the current induced for d-xylose in wt XylE (Fig. 6 and Table 3). The mutation enables the transporter to couple the proton translocation to the sugar release. Interestingly, wt XylE shows a higher apparent affinity for d-glucose compared with Q175I/L297F XylE (Table 3). As already indicated by the energy profile (Fig. 8), a lower *K*_*D*_ usually correlates with lower *V*_max_ because of an increase of the energy barrier for the formation of the occluded state.

As shown in Figure 4*C*, the higher apparent affinity for d-glucose compared with d-xylose is attained by a reduced rate for glucose release. Low *k*_off_ values are a common mechanism for inhibitors. The analysis of the interaction network for XylE bound to d-glucose explains the mechanism of inhibition by higher number of interactions between the residues likely leading to lower flexibility within the transporter compared with XylE bound to d-xylose (Fig. 7). These findings are also in agreement with a recent molecular dynamics study revealing that a d-glucose molecule within the XylE-binding pocket has a lower degree of motion compared with d-xylose (66).

### Conclusion

Substrate recognition by transporters represents the entry point for regulation of an organism's metabolism. We conclude that evolution changed the transport properties of MFS sugar/H^+^ symporters in a way to optimize for the transport of their major substrates, by increasing either apparent affinity (*K*_*M*_) or transport rate (*V*_max_). Increasing *V*_max_ is beneficial for major substrates with higher availability (*e.g.*, lactose transport in LacY) compared with substrates with lower physiological substrate concentrations (*e.g.*, fucose transport in FucP), where higher apparent affinity leads to improved transport rates. This statement seems trivial; however, our findings presented here strongly suggest that substrate specificity extends beyond the level of the substrate-binding site. In this view, the “Lock and Key” analogy, first postulated in 1894 by Emil Fischer, applies where the key (substrate) binds to the lock (transporter) pushing the pins (side-chain arrangement) in a way that releases the conformational confinement. In this way, the transporters may satisfy high substrate specificity on one hand, and on the other hand high conformational flexibility to convert the binding site to the other side of the membrane.

## Experimental procedures

### Plasmids and construction of mutants

Construction of the plasmids for pT7-5/wt LacY (18), pBAD-His A/wt FucP (67), and pET15b/wt XylE (68) has been described. Mutants for XylE and FucP were created by site-directed mutagenesis using the QuikChange Kit.

### Protein purification

Wt LacY and E325A LacY were purified from *E. coli* XL1-Blue cells (LB media) transformed with pT7-5 plasmids harboring the appropriate lacY gene by using Co(II) affinity chromatography as described (18).

Wt FucP, wt XylE, and given mutants were purified from *E. coli* BL21 DE3. Cells were grown in 2YT media at 37 °C, followed by induction at an absorbance of 0.8 at 600 nm with 0.2 mM IPTG (XylE) or 0.02% (w/v) arabinose (FucP), respectively, and growth was continued at 37 °C for 3 h. The cells were disrupted by a microfluidizer at 12.000 Psi followed by low-speed centrifugation. The supernatant was used for ultracentrifugation to harvest the membranes that were frozen and stored at −80 °C.

Membranes were solubilized at 5 mg/ml total protein in 50 mM sodium phosphate, NaPi (pH 7.5) containing 200 mM NaCl, 5 mM imidazole, a protease inhibitor cocktail tablet, and 1% *n*-dodecyl-beta-d-maltoside (w/v) (DDM) on ice. After centrifugation for 1 h (100,000*g* at 4 °C), the supernatant was used for purification of the His-tagged proteins by nickel–nitrilotriacetic acid affinity chromatography. After loading the sample and washing with 5 mM and 30 mM imidazole in 50 mM NaPi at pH 7.5 and 0.01% (w/v) DDM, purified proteins were eluted with 200 mM imidazole in the same buffer and 0.01% (w/v) DDM.

The eluted sample (10 ml) was concentrated to 2 to 5 mg/ml, final concentration, by using a concentrator with a 10 kDa cut off. At the same time, the buffer was exchanged with the buffer used for reconstitution (100 mM KPi, pH 7.5, and 2 mM MgSO_4_). The final yield was about 2 to 4 mg of wt protein and 0.2 to 1 mg of mutant protein per liter culture. The concentrated sample was used directly for reconstitution.

### Reconstitution of proteoliposomes

Reconstitution of purified proteins (2–20 mg/ml) was carried out with *E. coli* phospholipids (Avanti Polar Lipids, *E. coli* polar lipid extract). Preformed liposomes (0.2–2 ml, 10 mg/ml) were dissolved in 1% (w/v) *n*-octyl-β-d-glucoside, and the protein suspension was mixed on ice to a concentration of 0.2 mg protein/mg lipid (LPR 5).

Wt XylE, wt FucP, and the mutant proteins were reconstituted using overnight incubation in 400 mg/ml BioBeads (SM-2 Adsorbent Media, Bio-Rad) at 4 °C. After reconstitution, the samples were diluted to 2.5 mg/ml lipid concentration, frozen in liquid nitrogen, and stored at −80 °C. For wt LacY and mutant proteins, this procedure was not successful. We, therefore, applied the previously established *n*-octyl-β-d-glucoside dilution method (1:100) (69).

### SSM-based electrophysiology

SSM measurements were performed as described (20, 23). After thawing the sample and sonication in a water bath, 30 μl of proteoliposomes (2.5 mg/ml lipid at LPR 5) was allowed to adsorb for 1 to 2 h to an octadecanethiol/phosphatidylcholine hybrid bilayer on a gold surface (the sensor).

For measurements, a single solution exchange configuration (23) was employed that consisted in three phases of 0.5 s duration each: Flow of nonactivating solution (NA), activating solution (A), and nonactivating solution (NA). Only the activating solution contained the sugar. When pH dependence is determined, pH of NA and A solutions during one experiment are kept constant. Between experiments using different pH values, the sensor was incubated at the new pH for 3 min to equilibrate the intraliposomal pH.

Instruments with different time resolution were used as required. The high time-resolution set up with a valveless-diverted fluidic geometry had a flow rate of about 0.5 ml/s and a time resolution of 5 ms. The low time-resolution set up had a flow rate of 2 ml/s and a time resolution of 15 ms (22). Currents were recorded throughout the entire time and amplified with a current amplifier set to a gain of 10^−9^ V/A and a rise time of 10 ms (low time-resolution set up) or 3 ms (high time-resolution set up).

All NA solutions were buffered in 100 mM KPi at a given pH value. NA solutions used with LacY contained 1 mM DTT in addition. A solutions were made from NA solutions by adding the respective sugar at a given concentration to induce the symport reaction. Sugar concentration jumps initiated sugar-proton symport, and charge transfer was measured by capacitive coupling. Therefore, only transient currents were recorded.

### Data analysis

Each dataset represented with error bars was generated by performing the same experiment on multiple sensors (n ≥ 3). Error bars represent standard deviations of the average value. When peak currents are averaged across sensors, datasets are normalized before averaging. When the protein catalyzes transport and PSS currents are negligible, the peak current of the transient represents a good approximation of the current generated by the steady-state symport activity of the transporter. In most cases, peak currents have been used to derive kinetic and thermodynamic parameters according to the equations given in Figure 2 (*K*_*M*_, *I*_max_ ∼ *V*_max_, Hill coefficient n), Figure 4 (*K*_*D*_ and rate constants for sugar binding *k*_on_ and release *k*_off_), and Figure 6 (p*K*_a_ for proton binding and p*K*_b_ for proton release). Reconstruction of transporter currents for analysis of biphasic currents has been performed as described previously (70). This is required when transport and PSS currents are observed at the same time.

### Analysis of the side-chain interaction networks

The contact (adjacency) lists were generated analyzing the respective structure coordinate files (Protein Data Bank files) for an atom Van der Waals overlap of –0.4 Å with UCSF Chimera (71). Contacts with ≤3 bounds apart were discarded as well as connections of atoms with themselves. The network is represented by the adjacency matrix, where the elements of the matrix indicate whether pairs of atoms are in contact (adjacent) or not in the graph. Atoms belonging to the same residue, that is, side chain or ligand, were grouped, and the interactions were summed. The locally run Gephi script (version 0.8.2b and 0.9.1) (M. Bastian *et al.*, unpublished results) was used to construct the mathematical structures employed to model pairwise relations between the side chains or ligands in the respective proteins. In short, the statistics and metrics framework of the resulting contact graphs were calculated as a network with N nodes represented by a N × N binary matrix A = {*a*_*ij*_}, the adjacency matrix, whose element *a*_ij_ equals 1, when a link joining node *i* to node *j* is present and 0 otherwise (*i, j* = 1, 2, …, N). The degree *k*_i_ of a node *i* is defined as the number of links incident to node I:

$$k_{i}=\sum_{j}a_{ij}$$

In order to attach a quality measure (“*intensity*”) to specific interactions, weights of the links between nodes were introduced (weighted networks). The “intensity” *w*_*ij*_ of an interaction between two residues *i* and *j* is defined as:

$$w_{ij}=\underset{p}{\sum }\frac{\delta_{i}^{p}\delta_{j}^{p}}{n_{p}-1}\;if\;i\neq j,\;w_{ii}=0$$

where the index *p* runs over all residues in the protein, *n*_*p*_ is the number of contacts of residue *p*, and δ_*i*_^*p*^ is one if residue *i* has contributed an interaction to residue *p* and 0 otherwise (72, 73). Similarly, to the not weighted networks, in the N × N matrix *W* = {*w*_*ij*_}, *w*_*ij*_ is 0 if the nodes *i* and *j* are not linked. The weighted degree of a node represents here the connections per node on the graph weighted by the sum of interactions. Consequently, the weighted degree distribution is the probability that some residue has interactions of *w*_*ij*_ “*intensity*” to other neighboring residue.

The determination of the most central position in a network is a major issue in system characterization (74). An intuitive measure of the centrality is the degree of the node since more connected nodes are more central. However, this parameter disregards that nodes with low degree may be very critical by connecting different domains of the network. This attribute is quantified by counting the number of shortest paths between pairs of vertices that pass through a given vertex and is defined as BC (72, 73, 74, 75) of a node v, given by the expression:

$$g\left(v\right)=\sum_{i\neq v\neq j}\frac{\sigma_{ij}\left(v\right)}{\sigma_{ij}}$$

In this context, BC equals the sum of shortest paths (*σ*_*ij*_) connecting all nodes (side chains) to all others that involve the respective node (side chain). It measures the ranking of the side chain by the involvement in transferring information (*i.e.*, conformational forces) through the network. In different conformations of the protein, a specific side chain may be differently involved into the interaction network. To indicate those changes, BC alternations of respective residues are shown as the BC difference (ΔBC) of residues in the respective state of the transporter (Fig. 7).

## Data availability

The authors confirm that the data supporting the findings of this study are available within the article.

## Conflict of interest

The authors declare that they have no conflicts of interest with the contents of this article.
